# Associations of Parental Marijuana Use With Offspring Marijuana, Tobacco, and Alcohol Use and Opioid Misuse

**DOI:** 10.1001/jamanetworkopen.2019.16015

**Published:** 2019-11-22

**Authors:** Bertha K. Madras, Beth Han, Wilson M. Compton, Christopher M. Jones, Elizabeth I. Lopez, Elinore F. McCance-Katz

**Affiliations:** 1 McLean Hospital, Belmont, Massachusetts; 2 Department of Psychiatry, Harvard Medical School, Boston, Massachusetts; 3 Substance Abuse and Mental Health Services Administration, Rockville, Maryland; 4 National Institute on Drug Abuse, National Institutes of Health, Bethesda, Maryland; 5 Centers for Disease Control and Prevention, Atlanta, Georgia

## Abstract

**Importance:**

Marijuana use is increasing among adults and often co-occurs with other substance use; therefore, it is important to examine whether parental marijuana use is associated with elevated risk of substance use among offspring living in the same household.

**Objective:**

To examine associations of parental marijuana use with offspring marijuana, tobacco, and alcohol use and opioid misuse.

**Design, Setting, and Participants:**

This cross-sectional study used survey data from the 2015 through 2018 National Surveys on Drug Use and Health (NSDUH), which provide nationally representative data on adolescents or young adults living with a parent (the mother or the father). Annual average percentages were based on survey sampling weights. Final analyses were conducted September 21 through 23, 2019.

**Exposures:**

Parental marijuana use status.

**Main Outcomes and Measures:**

Offspring self-reported use of marijuana, tobacco, or alcohol or misuse of opioids.

**Results:**

Survey respondents included 24 900 father-offspring or mother-offspring dyads sampled from the same household. Among mothers living with adolescent offspring, 8.2% (95% CI, 7.3%-9.2%) had past-year marijuana use, while 7.6% (95% CI, 6.2%-9.2%) of mothers living with young adult offspring had past-year marijuana use. Among fathers living with adolescent offspring, 9.6% (95% CI, 8.5%-10.8%) had past-year marijuana use, and 9.0% (95% CI, 7.4%-10.9%) of fathers living with young adult offspring had past-year marijuana use. Compared with adolescents whose mothers never used marijuana, adjusted relative risk (ARR) of past-year marijuana use was higher among those whose mothers had lifetime (without past-year) marijuana use (ARR, 1.3; 95% CI, 1.1-1.6; *P* = .007), less than 52 days of past-year marijuana use (ARR, 1.7; 95% CI, 1.1-2.7; *P* = .02), or 52 days or more of past-year marijuana use (ARR, 1.5; 95% CI, 1.1-2.2; *P* = .02). Compared with young adults whose mothers never used marijuana, adjusted risk of past-year marijuana use was higher among those whose mothers had lifetime (without past-year) marijuana use (ARR, 1.4; 95% CI, 1.1-1.7; *P* = .001), less than 52 days of past-year marijuana use (ARR, 1.5; 95% CI, 1.0-2.3; *P* = .049), or 52 days or more of past-year marijuana use (ARR, 1.8; 95% CI, 1.3-2.5; *P* = .002). Compared with adolescents whose fathers never used marijuana, adolescents whose fathers had less than 52 days of past-year marijuana use were more likely to use marijuana (ARR, 1.8; 95% CI, 1.2-2.7; *P* = .006). Compared with young adults whose fathers never used marijuana, young adults whose fathers had 52 days or more of past-year marijuana use were more likely to use marijuana (ARR, 2.1; 95% CI, 1.6-2.9; *P* < .001). Compared with their peers whose parents never used marijuana and after adjusting for covariates, the adjusted risk of past-year tobacco use was higher among adolescents whose mothers had lifetime marijuana use (ARR, 1.3; 95% CI, 1.0-1.6; *P* = .03), less than 52 days of past-year marijuana use (ARR, 1.5; 95% CI, 1.0-2.1; *P* = .04), or 52 days or more of past-year marijuana use (ARR, 1.6; 95% CI, 1.1-2.3; *P* = .03); adolescents whose fathers had lifetime marijuana use (ARR, 1.5; 95% CI, 1.1-1.9; *P* = .004) or 52 days or more of past-year marijuana use (ARR, 1.8; 95% CI, 1.2-2.7; *P* = .006); young adults whose mothers had lifetime marijuana use (ARR, 1.2; 95% CI, 1.0-1.4; *P* = .04); and young adults whose fathers had 52 days or more of past-year marijuana use (ARR, 1.4; 95% CI, 1.0-1.9; *P* = .046). Compared with their peers whose parents had no past marijuana use and after adjusting for covariates, risk of past-year alcohol use was higher among adolescents whose mothers had lifetime marijuana use (ARR, 1.2; 95% CI, 1.1-1.4; *P* = .004), less than 52 days of past-year marijuana use (ARR, 1.5; 95% CI, 1.2-1.9; *P* = .002), or 52 days or more of past-year marijuana use (ARR, 1.3; 95% CI, 1.0-1.7; *P* = .04). After adjusting for covariates, parental marijuana use was not associated with opioid misuse by offspring.

**Conclusions and Relevance:**

In this cross-sectional study, parental marijuana use was associated with increased risk of substance use among adolescent and young adult offspring living in the same household. Screening household members for substance use and counseling parents on risks posed by current and past marijuana use are warranted.

## Key Points

**Question:**

Is parental marijuana use associated with marijuana, tobacco, and alcohol use and opioid misuse by adolescent and young adult offspring living in the same household?

**Findings:**

In this cross-sectional study including 24 900 father-offspring or mother-offspring dyads, parental marijuana use was associated with increased risk of marijuana and tobacco use and opioid misuse by both adolescent and young adult offspring and of alcohol use by adolescent offspring.

**Meaning:**

Screening household members for substance use and counseling parents on risks posed by current and past marijuana use may be helpful in the effort to prevent a cycle of multigenerational substance use.

## Teaser

This cross-sectional study examines the associations of parental marijuana use with marijuana, tobacco, and alcohol use and opioid misuse in their adolescent or young adult offspring.

## Introduction

The shifting legal environment for marijuana in the United States is traceable to a 1996 California ballot initiative that approved use of marijuana for various medical conditions. Thereafter, medicalization proliferated in other states and spawned a derivative movement to legalize nonmedical use of marijuana. With legal status and social acceptability of marijuana ascendant, the marijuana industry has created products to enhance palatability and potency^1^ and expand modes of delivery.^2^ One outcome of this shift in marijuana status is an overall increase in past-year marijuana use from 25.8 million people (11.0%) in 2002 to 43.5 million (15.9%) in 2018 among US people aged 12 years or older, with noteworthy increases in young adults.^3^

Paralleling these trends is increasing apprehension of the health consequences of marijuana, especially among adolescents.^4^ Early marijuana initiation is associated with higher rates of addiction,^5^ impaired cognition,^6,7^ preclinical or clinical symptoms of psychosis, schizophrenia, depression, suicidality,^8,9,10,11^ and reduced educational achievement^12^ and employment status.^13^ Peer influence, genetics, family environment, family interactions, and quality of parenting are among the risk factors and protective modulators of children’s substance use.^14,15,16,17^

With peak marijuana use occurring among adults of childbearing and childrearing ages, parental marijuana use conceivably poses a direct environmental risk of normalizing marijuana use and enabling access to marijuana for their offspring. Adolescent marijuana use is highest among those with parents and peers who use marijuana compared with nonusing counterparts,^18^ whereas peer influence on youth substance use can be neutralized by parents who do not use substances.^19^ In general, living with a parent who uses substances or has substance use disorder is a risk factor for use of substances among young offspring.^20,21^ Yet, few studies have directly examined whether parental marijuana use increases the risk of opioid misuse among adolescent and young adult offspring living in the same household, a critical gap in view of the current opioid crisis.^22^ Most importantly, none of the existing epidemiological studies simultaneously examined associations between parental marijuana use at detailed frequency levels and adolescent and young adult offspring’s marijuana, tobacco, and alcohol use, and opioid misuse, to our knowledge.

To address this gap, this study examined potential intergenerational associations within specific substances and across substances. Specifically, we examined whether parental marijuana use was associated with offspring marijuana, tobacco, or alcohol use or opioid misuse. Adolescents and young adults have distinct developmental stages, so separate analyses were conducted among adolescent and young adult offspring. Our results could help to inform clinicians, parents, and policy makers regarding substance use prevention.

## Methods

### Data Source

We examined data from the 2015 through 2018 National Surveys on Drug Use and Health (NSDUH), conducted by the Substance Abuse and Mental Health Services Administration (SAMHSA). The NSDUH data collection protocol was approved by the institutional review board at RTI International.^23^ Since this was a minimal risk survey, it met institutional review board requirements for a waiver of written informed consent. Verbal informed consent was received from each participant.

The NSDUH used a stratified, multistage area probability sample that was designed to be nationally representative on substance use among the US civilian, noninstitutionalized population 12 years or older. Participants included adolescents aged 12 to 17 years living with a sampled parent who was born from 1955 to 1984 and young adults aged 18 to 30 years living with a sampled parent who was born from 1955 to 1980. After dwelling unit (DU) selections were made, an interviewer visited each selected DU to obtain a roster of all people residing in the DU. A maximum of 2 people were selected from any DU, so the NSDUH never sampled a child and both parents within each sampled DU.^24^ The subsamples examined in this study are weighted to be nationally representative of an offspring (adolescent or young adult) residing with their mother or father.

Interviews lasted approximately 1 hour. Audio computer-assisted self-administered interviewing was used, providing a private, confidential way to record answers. The annual mean (SD) weighted response rate for the 2015 through 2018 NSDUH was 51.9% (2.9%). Details regarding NSDUH methods and NSDUH pair samples are provided elsewhere.^23,24^

### Measures

The NSDUH collected data on lifetime use (yes or no) and past-year use (yes or no) of tobacco, alcohol, and illicit drugs (ie, marijuana, cocaine, heroin, hallucinogens, inhalants, and methamphetamine), and misuse (yes or no) of prescription psychotherapeutics (opioids, stimulants, sedatives, and tranquilizers). Among past-year marijuana users, the NSDUH assessed the number of days of marijuana use in the past year. This study examined parental marijuana use status at 4 levels: never use, lifetime (without past-year) use, less than 52 days of past-year use, and 52 days or more of past-year use (ie, approximately weekly or more).

The opioid misuse category (yes or no) included heroin use and prescription opioid misuse. Prescription opioid misuse was defined as use in any way that a doctor did not direct the respondent, including use without the respondent's own prescription; use in greater amounts, more often, or longer than the respondent was told; or use in any other way a doctor did not direct the respondent.^25^

Among respondents with lifetime use of marijuana, tobacco, or alcohol, the NSDUH collected their age at initiation (first time ever used).^5,26^ Among respondents reporting past-month alcohol use, NSDUH also collected past-month binge alcohol use (yes or no), defined as drinking 5 or more drinks for men or 4 or more drinks for women on the same occasion on at least 1 day in the past month.

When assessing associations of parental marijuana use and adolescent offspring substance use, we controlled for the corresponding substance use of adolescent’s peers and parenting styles associated with adolescent substance use.^14,15,16,17,18^ For adolescent respondents, the NSDUH assessed how many of an adolescent’s peers used marijuana, alcohol, or tobacco (none, a few, most, or all), the adolescent’s perceived parental disapproval of using marijuana once a month or more, perceived parental disapproval of drinking alcohol daily, and perceived parental disapproval of smoking one pack of cigarettes per day (parental disapproval measures: neither approve nor disapprove, somewhat disapprove, or strongly disapprove).^26,27^

Offspring substance use may be associated with parental mental health status^14,15,16,17^ and offspring’s depression,^8,9,10,11^ which we controlled for when examining associations of parental marijuana use with offspring substance use. The NSDUH provided estimates of past-year major depressive episode (yes or no) among adolescent and adult respondents based on assessments of individual diagnostic criteria from the *Diagnostic and Statistical Manual of Mental Disorders* (Fourth Edition) (*DSM-IV*),^28^ which has demonstrated good reliability and validity.^29,30^ Past-year mental illness status (ie, having had a diagnosable mental disorder [excluding developmental disorders and substance use disorders] of sufficient duration to meet diagnostic criteria specified within the *DSM-IV* [yes or no]) was determined for each adult respondent using a predictive model based on questions about distress (past-year K6 scale), impairment (truncated version of the World Health Organization Disability Assessment Schedule), serious suicidal ideation, major depressive episode, and age.^31,32^

A state’s medical or nonmedical marijuana use legal status and urban or rural residence location are associated with young people’s substance use.^26^ Using state and year information, we created a time-dependent variable indexing state legalization of medical and nonmedical marijuana use (yes or no). Additionally, the NSDUH collected information on respondents’ age, sex, race/ethnicity, family income, and metropolitan statistical area (MSA) (large, small, or non-MSA). Race/ethnicity was based on the respondent’s self-classification of racial and ethnic and identification and origin based on the classifications developed by the US Census Bureau.

### Statistical Analysis

First, we estimated the prevalence of parental marijuana use and mean initiation ages. Second, we estimated the prevalence of past-year use of marijuana, tobacco, and alcohol and past-year opioid misuse among adolescents living with a parent by parental marijuana use status. Similarly, we estimated the prevalence of past-year marijuana and tobacco use, past-year opioid misuse, and past-month binge alcohol use among young adults living with a parent by parental marijuana use status.

Bivariable and multivariable logistic regression models were applied to examine the associations of parental marijuana use with substance use of adolescent and young adult offspring separately. Unadjusted relative risks (RRs) and adjusted RRs (ARRs) were estimated using PREDMARG and PRED_EFF statements in SUDAAN statistical software version 11.0.1 (RTI International).^33^ *P* values were calculated using 2-sided *t* tests, and statistical significance was set at less than .05. Multivariable models included covariates for offspring (age, sex, race/ethnicity, past-year major depressive episode, and perceived parental disapproval of adolescent substance use), family (income and parental mental illness status), environment (peer substance use, state’s legal status of medical and nonmedical marijuana, and MSA), and parental tobacco use and nonmarijuana illicit drug use. We additionally controlled for parental alcohol use status when examining for correlates of offspring alcohol use and binge alcohol use. Multicollinearity and potential interaction effects between examined factors were assessed and were not identified in final multivariable models. SUDAAN software version 11.0.1 was used for all analyses to account for the complex sample design and sampling weights of NSDUH data. Final analyses were conducted September 21 through 23, 2019.

## Results

Among parents born from 1955 to 1984 living with adolescent offspring, 8.2% (95% CI, 7.3%-9.2%) of mothers and 9.6% (95% CI, 8.5%-10.8%) of fathers had used marijuana in the past year, and 3.5% (95% CI, 3.0%-4.1%) of mothers and 5.4% (95% CI, 4.6%-6.2%) of fathers had used marijuana on 52 days or more in the past year (Table 1). Among parents with lifetime marijuana use, mean marijuana initiation age was 18.0 (95% CI, 17.8-18.2) years among mothers and 17.2 (95% CI, 17.0-17.5) years among fathers. Among parents born from 1955 to 1980 living with young adult offspring, 7.6% (95% CI, 6.2%-9.2%) of mothers and 9.0% (95% CI, 7.4%-10.9%) of fathers had used marijuana in the past year, and 3.7% (95% CI, 2.8%-4.7%) of mothers and 5.4% (95% CI, 4.1%-7.0%) of fathers had used marijuana on 52 days or more in the past year. Among parents of young adult offspring with lifetime marijuana use, mean marijuana initiation age was 18.2 (95% CI, 17.7-18.7) years among mothers and 17.2 (95% CI, 16.8-17.6) years among fathers.

**Table 1.** Parental Marijuana Use Status and Mean Age of Initiation In Parents With Lifetime Marijuana Use

Parental Marijuana Use	Weighted % (95% CI)			
	Mothers Born in 1955-1984 Living With Offspring Aged 12-17 y (n = 11 500^a^)	Fathers Born in 1955-1984 Living With Offspring Aged 12-17 y (n = 7200^a^)	Mothers Born in 1955-1980 Living With Offspring Aged 18-30 y (n = 4000^a^)	Fathers Born in 1955-1980 Living With Offspring Aged 18-30 y (n = 2300^a^)
Never use	56.3 (54.8-57.8)	49.0 (47.0-51.0)	57.9 (55.2-60.6)	52.7 (49.3-56.3)
Lifetime use but not past y use	35.5 (34.1-37.0)	41.4 (39.5-43.3)	34.5 (31.9-37.1)	38.3 (35.0-41.7)
Any use in past y	8.2 (7.3-9.2)	9.6 (8.5-10.8)	7.6 (6.2-9.2)	9.0 (7.4-10.9)
<52 d use in past y	4.7 (4.0-5.5)	4.2 (3.5-5.1)	3.9 (2.9-5.3)	3.6 (2.7-4.8)
≥52 d use in past y	3.5 (3.0-4.1)	5.4 (4.6-6.2)	3.7 (2.8-4.7)	5.4 (4.1-7.0)
Age at marijuana initiation, mean (95% CI), y	18.0 (17.8-18.2)	17.2 (17.0-17.5)	18.2 (17.7-18.7)	17.2 (16.8-17.6)

^a^ Substance Abuse and Mental Health Services Administration requires that any description of overall sample sizes based on the restricted-use National Surveys on Drug Use and Health data files be rounded to the nearest 100 to minimize potential disclosure risk.

### Adolescent Offspring Marijuana Use by Parental Marijuana Use Status 

Compared with adolescents whose parents never used marijuana, the unadjusted risk of past-year marijuana use was higher among adolescents whose mothers had lifetime marijuana use (RR, 1.9; 95% CI, 1.6-2.2; *P* < .001), less than 52 days of past-year marijuana use (RR, 2.5; 95% CI, 1.8-3.6; *P* < .001), or 52 days or more of past-year marijuana use (RR, 2.5; 95% CI, 1.9-3.4; *P* < .001) and among adolescents whose fathers had lifetime marijuana use (RR, 2.0; 95% CI, 1.6-2.5; *P* < .001), less than 52 days of past-year marijuana use (RR, 3.7; 95% CI, 2.4-5.7; *P* < .001), or 52 days or more of past-year marijuana use (RR, 2.6; 95% CI, 1.8-3.7; *P* < .001) (Table 2). Compared with adolescents whose mothers never used marijuana, the adjusted risk of past-year marijuana use was higher among adolescents whose mothers had lifetime (without past-year) marijuana use (ARR, 1.3; 95% CI, 1.1-1.6; *P* = .007), less than 52 days of past-year marijuana use (ARR, 1.7; 95% CI, 1.1-2.7; *P* = .02), and or 52 days or more of past-year marijuana use (ARR, 1.5; 95% CI, 1.1-2.2; *P* = .02). The adjusted risk of past-year marijuana use was higher among adolescents whose fathers had less than 52 days of past-year marijuana use (ARR, 1.8; 95% CI, 1.2-2.7; *P* = .006) than among adolescents whose fathers never used marijuana (Table 2).

**Table 2.** Past-Year Marijuana Use and Opioid Misuse Among Adolescent Offspring Aged 12 to 17 Years Who Lived With a Parent Born in 1955 to 1984 by Parental Marijuana Use Status (n = 37 300)

Parental Marijuana Use Status	Marijuana Use, Past y			Heroin Use or Prescription Opioid Misuse, Past y		
	% (SE)	RR (95% CI)	ARR (95% CI)^a^	% (SE)	RR (95% CI)	ARR (95% CI)^b^
Mother						
Never use	8.4 (0.6)	1 [Reference]	1 [Reference]	2.5 (0.3)	1 [Reference]	1 [Reference]
LT use, not in in past y	15.6 (0.8)	1.9 (1.6-2.2)	1.3 (1.1-1.6)	4.0 (0.5)	1.6 (1.1-2.3)	1.4 (0.9-2.4)
<52 d use in past y	21.3 (3.5)	2.5 (1.8-3.6)	1.7 (1.1-2.7)	6.7 (2.4)	2.7 (1.3-5.7)	2.5 (1.0-6.4)
≥52 d use in past y	21.2 (2.9)	2.5 (1.9-3.4)	1.5 (1.1-2.2)	5.0 (1.7)	2.0 (1.0-3.9)	1.8 (0.8-3.8)
Father						
Never use	6.8 (0.7)	1 [Reference]	1 [Reference]	1.7 (0.3)	1 [Reference]	1 [Reference]
LT use, not in past y	13.3 (1.1)	2.0 (1.6-2.5)	1.2 (1.0-1.5)	3.2 (0.6)	1.8 (1.1-3.1)	1.3 (0.8-2.3)
<52 d use in past y	25.1 (4.7)	3.7 (2.4-5.7)	1.8 (1.2-2.7)	6.1 (2.1)	3.5 (1.7-7.4)	2.1 (0.9-4.9)
≥52 d use in past y	17.5 (2.5)	2.6 (1.8-3.7)	1.3 (0.9-1.9)	4.0 (1.2)	2.3 (1.2-4.6)	1.2 (0.6-2.5)

Abbreviations: ARR, adjusted relative risk; LT, lifetime; RR, relative risk.

^a^ Model controlled for adolescents’ age, sex, race/ethnicity, family income, past-year major depressive episode, marijuana use of peers, perceived parent disapproval of using marijuana once a month or more, metropolitan statistical area, state’s medical and nonmedical marijuana use legal status, father’s or mother’s mental illness status and lifetime and past-year tobacco use, nonmarijuana illicit drug use, and misuse of prescription psychotherapeutics, and survey year.

^b^ Controlled for all the covariates above except for marijuana use of adolescents’ peers, adolescents’ perceived parent disapproval of using marijuana once a month or more.

### Adolescent Offspring Opioid Misuse by Parental Marijuana Use Status

Compared with adolescents whose parents never used marijuana, the unadjusted risk of past-year opioid misuse was higher among adolescents whose mothers had lifetime marijuana use (RR, 1.6; 95% CI, 1.1-2.3; *P* = .006), less than 52 days of past-year marijuana use (RR, 2.7; 95% CI, 1.3-5.7; *P* = .01), or 52 days or more of past-year marijuana use (RR, 2.0; 95% CI, 1.0-3.9; *P* = .05) and among adolescents whose fathers had lifetime marijuana use (RR, 1.8; 95% CI, 1.1-3.1; *P* = .02), less than 52 days of past-year marijuana use (RR, 3.5; 95% CI, 1.7-7.4; *P* = .001), or 52 days or more of past-year marijuana use (RR, 2.3; 95% CI, 1.2-4.6; *P* = .01) (Table 2). However, after controlling for covariates (excluding marijuana use of adolescents’ peers and adolescents’ perceived parental disapproval of using marijuana), the adjusted risk of past-year opioid misuse by adolescent offspring did not vary by parental marijuana use status.

### Adolescent Offspring Tobacco Use by Parental Marijuana Use Status

Compared with adolescents whose parents never used marijuana, the unadjusted risk of past-year tobacco use was higher among adolescents whose mothers had lifetime marijuana use (RR, 1.9; 95% CI, 1.6-2.3; *P* < .001), less than 52 days of past-year marijuana use (RR, 2.0; 95% CI, 1.4-3.1; *P* = .001), or 52 days or more of past-year marijuana use (RR, 2.2; 95% CI, 1.5-3.3; *P* < .001) and among adolescents whose fathers had lifetime marijuana use (RR, 2.1; 95% CI, 1.6-2.7; *P* < .001), less than 52 days of past-year marijuana use (RR, 1.8; 95% CI, 1.1-3.0; *P* = .03), or 52 days or more of past-year marijuana use (RR, 2.8; 95% CI, 2.0-2.7; *P* < .001) (Table 3). The multivariable model controlled for the same covariates as the model for adolescent offspring opioid misuse, plus cigarette use by adolescents’ peers and adolescents’ perceived parental disapproval of smoking. Compared with adolescents whose mothers never used marijuana, the adjusted risk of past-year tobacco use was higher among adolescents whose mothers had lifetime marijuana use (ARR, 1.3; 95% CI, 1.0-1.6; *P* = .03), less than 52 days of past-year marijuana use (ARR, 1.5; 95% CI, 1.0-2.1; *P* = .04), or 52 days or more of past-year marijuana use (ARR, 1.6; 95% CI, 1.1-2.3; *P* = .03). Compared with adolescents whose fathers never used marijuana, the adjusted risk of past-year tobacco use was higher among adolescents whose fathers had lifetime marijuana use (ARR, 1.5; 95% CI, 1.1-1.9; *P* = .004) or 52 days or more of past-year marijuana use (ARR, 1.8; 95% CI, 1.2-2.7; *P* = .006) (Table 3).

**Table 3.** Past-Year Tobacco and Alcohol Use Among Adolescent Offspring Aged 12 to 17 Years Who Lived With a Parent Born in 1955 to 1984 by Parental Marijuana Use Status (n = 37 300)

Parental Marijuana Use Status	Tobacco Use, Past y			Alcohol Use, Past y		
	% (SE)	RR (95% CI)	ARR (95% CI)^a^	% (SE)	RR (95% CI)	ARR (95% CI)^b^
Mother						
Never use	6.1 (0.5)	1 [Reference]	1 [Reference]	16.1 (0.8)	1 [Reference]	1 [Reference]
LT use, not in past y	11.8 (0.8)	1.9 (1.6-2.3)	1.3 (1.0-1.6)	25.8 (1.1)	1.6 (1.4-1.8)	1.2 (1.1-1.4)
<52 d use in past y	12.5 (2.4)	2.0 (1.4-3.1)	1.5 (1.0-2.1)	28.4 (3.5)	1.8 (1.4-2.3)	1.5 (1.2-1.9)
≥52 d use in past y	13.7 (2.5)	2.2 (1.5-3.3)	1.6 (1.1-2.3)	25.4 (3.1)	1.6 (1.2-2.0)	1.3 (1.0-1.7)
Father						
Never use	5.5 (0.5)	1 [Reference]	1 [Reference]	15.1 (1.0)	1 [Reference]	1 [Reference]
LT use, not in past y	11.6 (1.0)	2.1 (1.6-2.7)	1.5 (1.1-1.9)	23.6 (1.3)	1.6 (1.3-1.9)	1.1 (0.9-1.3)
<52 d use in past y	9.8 (2.3)	1.8 (1.1-3.0)	1.0 (0.6-1.8)	34.2 (4.9)	2.3 (1.7-3.1)	1.3 (0.9-1.9)
≥52 d use in past y	15.7 (2.5)	2.8 (2.0-4.1)	1.8 (1.2-2.7)	25.0 (3.2)	1.7 (1.3-2.2)	1.3 (1.0-1.8)

Abbreviations: ARR, adjusted relative risk; LT, lifetime; RR, relative risk.

^a^ Model controlled for adolescents’ age, sex, race/ethnicity, family income, past-year major depressive episode, metropolitan statistical area, state’s medical and nonmedical marijuana use legal status, father’s or mother’s mental illness status and lifetime and past-year tobacco use, nonmarijuana illicit drug use, and misuse of prescription psychotherapeutics, survey year, cigarette use of adolescents’ peers, and adolescents’ perceived parent disapproval of smoking 1 pack of cigarettes per day.

^b^ Model controlled for adolescents’ age, sex, race/ethnicity, family income, past-year major depressive episode, metropolitan statistical area, state’s medical and nonmedical marijuana use legal status, father’s or mother’s mental illness status and lifetime and past-year tobacco use, nonmarijuana illicit drug use, and misuse of prescription psychotherapeutics, survey year, alcohol use of adolescents’ peers, adolescents’ perceived parent disapproval of drinking alcohol daily, and father’s or mother’s lifetime and past-year alcohol use status.

### Adolescent Offspring Alcohol Use by Parental Marijuana Use Status

Compared with adolescents whose parents never used marijuana, the unadjusted risk of past-year alcohol use was higher among adolescents whose mothers had lifetime marijuana use (RR, 1.6; 95% CI, 1.4-1.8; *P* < .001), less than 52 days of past-year marijuana use (RR, 1.8; 95% CI, 1.4-2.3; *P* < .001), or 52 days or more of past-year marijuana use (RR, 1.6; 95% CI, 1.2-2.0; *P* < .001) and among adolescents whose fathers had lifetime marijuana use (RR, 1.6; 95% CI, 1.3-1.9; *P* < .001), less than 52 days of past-year marijuana use (RR, 2.3; 95% CI, 1.7-3.1; *P* < .001), or 52 days or more of past-year marijuana use (RR, 1.7; 95% CI, 1.3-2.2; *P* < .001) (Table 3). The multivariable model included the same covariates as in the model for adolescent offspring opioid misuse, plus alcohol use of adolescents’ peers, adolescents’ perceived parental disapproval of daily alcohol use, and parental alcohol use status. Compared with adolescents whose mother never used marijuana, the adjusted risk of past-year alcohol use was higher among adolescents whose mothers had lifetime marijuana use (ARR, 1.2; 95% CI, 1.1-1.4; *P* = .004), less than 52 days of past-year marijuana use (ARR, 1.5; 95% CI, 1.2-1.9; *P* = .002), or 52 days or more of past-year marijuana use (ARR, 1.3; 95% CI, 1.0-1.7; *P* = .04) (Table 3). The adjusted risk of past-year alcohol use among adolescent offspring did not vary by father’s marijuana use status.

### Young Adult Offspring’s Marijuana Use by Parental Marijuana Use Status 

Compared with young adults whose parents never used marijuana, the unadjusted risk of past-year marijuana use was higher among young adults whose mother had lifetime marijuana use (RR, 1.8; 95% CI, 1.5-2.1; *P* < .001), less than 52 days of past-year marijuana use (RR, 2.2; 95% CI, 1.6-3.1; *P* < .001), or 52 days or more of past-year marijuana use (RR, 2.7; 95% CI, 2.1-3.4; *P* < .001) and among young adults whose fathers had lifetime marijuana use (RR, 1.5; 95% CI, 1.2-1.9; *P* < .001), less than 52 days of past-year marijuana use (RR, 2.0; 95% CI, 1.4-2.9; *P* = .002), or 52 days or more of past-year marijuana use (RR, 2.8; 95% CI, 2.1-3.7; *P* < .001) (Table 4). Compared with young adults whose mothers never used marijuana, the adjusted risk of past-year marijuana use was higher among those whose mothers had lifetime marijuana use (ARR, 1.4; 95% CI, 1.1-1.7; *P* = .001), less than 52 days of past-year marijuana use (ARR, 1.5; 95% CI, 1.0-2.3; *P* = .049), or 52 days or more of past-year marijuana use (ARR, 1.8; 95% CI, 1.3-2.5; *P* = .002). The adjusted risk of past-year marijuana use was higher among young adults whose fathers had 52 days or more of past-year marijuana use (ARR, 2.1; 95% CI, 1.6-2.9; *P* < .001) compared with those whose fathers never used marijuana (Table 4).

**Table 4.** Past-Year Marijuana Use and Opioid Misuse Among Young Adult Offspring Aged 18-30 Years Who Lived With a Parent Born in 1955 to 1980 by Parental Marijuana Use Status (n = 12 600)

Parental Marijuana Use Status	Marijuana Use, Past y			Heroin Use or Prescription. Opioid Misuse, Past y		
	% (SE)	RR (95% CI)	ARR (95% CI)^a^	% (SE)	RR (95% CI)	ARR (95% CI)^a^
Mother						
Never use	22.7 (1.5)	1 [Reference]	1 [Reference]	5.8 (0.8)	1 [Reference]	1 [Reference]
LT use, not in past y	41.2 (2.1)	1.8 (1.5-2.1)	1.4 (1.1-1.7)	9.4 (1.2)	1.6 (1.1-2.4)	1.3 (0.7-2.2)
<52 d use in past y	50.5 (7.8)	2.2 (1.6-3.1)	1.5 (1.0-2.3)	12.9 (5.8)	2.2 (0.9-5.6)	1.3 (0.5-3.7)
≥52 d use in past y	61.2 (6.4)	2.7 (2.1-3.4)	1.8 (1.3-2.5)	20.2 (5.1)	3.5 (2.0-6.2)	1.9 (0.8-4.6)
Father						
Never use	21.1 (1.8)	1 [Reference]	1 [Reference]	4.8 (0.8)	1 [Reference]	1 [Reference]
LT use, not in in past y	32.7 (2.6)	1.5 (1.2-1.9)	1.1 (0.9-1.4)	7.6 (1.3)	1.6 (1.0-2.5)	1.1 (0.6-2.0)
<52 d use in past y	41.9 (7.2)	2.0 (1.4-2.9)	1.4 (0.9-2.2)	9.2 (3.7)	1.9 (0.8-4.5)	1.4 (0.5-3.7)
≥52 d use in past y	58.9 (7.1)	2.8 (2.1-3.7)	2.1 (1.6-2.9)	10.5 (3.5)	2.2 (1.0-4.5)	1.7 (0.8-3.7)

Abbreviations: ARR, adjusted relative risk; LT, lifetime; RR, relative risk.

^a^ Controlled for young adult’s age, sex, race/ethnicity, family income, past-year major depressive episode, metropolitan statistical area, state’s medical and nonmedical marijuana use legal status, father’s or mother’s mental illness status and lifetime and past-year tobacco use, nonmarijuana illicit drug use, misuse of prescription psychotherapeutics, and survey year.

### Young Adult Offspring Opioid Misuse by Parental Marijuana Use Status

Compared with young adults whose parents never used marijuana, the unadjusted risk of past-year opioid misuse was higher among those whose mothers had 52 days or more of past-year marijuana use (RR, 3.5; 95% CI, 2.0-6.2; *P* < .001) and among those whose fathers had 52 days or more of past-year marijuana use (RR, 2.2; 95% CI, 1.0-4.5; *P* = .04) (Table 4). After controlling for covariates, their opioid misuse did not vary by parental marijuana use status.

### Young Adult Offspring Tobacco Use by Parental Marijuana Use Status

Compared with young adults whose parents never used marijuana, the unadjusted risk of past-year tobacco use was higher among those whose mothers had lifetime marijuana use (RR, 1.5; 95% CI, 1.3-1.7; *P* < .001), less than 52 days of past-year marijuana use (RR, 1.5; 95% CI, 1.1-2.0; *P* = .02), or 52 days or more of past-year marijuana use (RR, 1.7; 95% CI, 1.3-2.3; *P* = .001) and among those whose fathers had lifetime marijuana use (RR, 1.4; 95% CI, 1.2-1.7; *P* < .001), less than 52 days of past-year marijuana use (RR, 1.9; 95% CI, 1.4-2.5; *P* = .001), or 52 days or more of past-year marijuana use (RR, 1.8; 95% CI, 1.4-2.5; *P* < .001) (Table 5). After controlling for the same covariates as in the multivariable model for young adult offspring marijuana use, risk of past-year tobacco use was higher among young adults whose mothers had lifetime marijuana use (ARR, 1.2; 95% CI, 1.0-1.4; *P* = .04) than among those whose mothers never used marijuana. The adjusted risk of past-year tobacco use was also higher among those whose fathers had 52 days or more of past-year marijuana use (ARR, 1.4; 95% CI, 1.0-1.9; *P* = .046) than among those whose fathers never used marijuana (Table 5).

**Table 5.** Past-Year Tobacco Use and Past-Month Binge Alcohol Use Among Young Adult Offspring Aged 18-30 Years Who Lived With a Parent Born in 1955 to 1980 by Parental Marijuana Use Status (n = 12 600)

Parental Marijuana Use Status	Tobacco Use, Past y			Binge Alcohol Use, Past mo.		
	% (SE)	RR (95% CI)	ARR (95% CI)^a^	% (SE)	RR (95% CI)	ARR (95% CI)^b^
Mother						
Never use	29.2 (1.5)	1 [Reference]	1 [Reference]	30.3 (1.6)	1 [Reference]	1 [Reference]
LT use, not in past y	43.6 (2.1)	1.5 (1.3-1.7)	1.2 (1.0-1.4)	36.7 (2.1)	1.2 (1.0-1.4)	1.0 (0.8-1.2)
<52 d use in past y	43.2 (6.0)	1.5 (1.1-2.0)	1.2 (0.8-1.7)	48.8 (6.9)	1.6 (1.2-2.2)	1.2 (0.9-1.7)
≥52 d use in past y	50.2 (6.6)	1.7 (1.3-2.3)	1.2 (0.9-1.8)	37.1 (6.0)	1.2 (0.9-1.7)	0.9 (0.6-1.4)
Father						
Never use	27.7 (2.1)	1 [Reference]	1 [Reference]	27.8 (2.2)	1 [Reference]	1 [Reference]
LT use, not in past y	39.5 (2.6)	1.4 (1.2-1.7)	1.1 (0.9-1.3)	43.4 (2.7)	1.6 (1.3-1.9)	1.3 (1.1-1.6)
<52 d use in past y	51.4 (7.4)	1.9 (1.4-2.5)	1.2 (0.8-1.8)	47.2 (7.2)	1.7 (1.2-2.4)	1.4 (1.0-2.1)
≥52 d use in past y	51.1 (6.9)	1.8 (1.4-2.5)	1.4 (1.0-1.9)	27.8 (5.7)	1.0 (0.7-1.5)	1.0 (0.6-1.5)

Abbreviations: ARR, adjusted relative risk; LT, lifetime; RR, relative risk.

^a^ Controlled for young adult’s age, sex, race/ethnicity, family income, past-year major depressive episode, metropolitan statistical area, state’s medical and nonmedical marijuana use legal status, father’s or mother’s mental illness status and lifetime and past-year tobacco use, nonmarijuana illicit drug use, misuse of prescription psychotherapeutics, and survey year.

^b^ Controlled for all of the above covariates and father’s or mother’s lifetime or past-year alcohol use status.

### Young Adult Offspring Binge Alcohol Use by Parental Marijuana Use Status

Compared with young adults whose parents never used marijuana, the unadjusted risk of past-month binge alcohol use was higher among young adults whose mothers had lifetime marijuana use (RR, 1.2; 95% CI, 1.0-1.4; *P* < .001) or less than 52 days of past-year marijuana use (RR, 1.6; 95% CI, 1.2-2.2; *P* = .008) and among those whose fathers had lifetime marijuana use (RR, 1.6; 95% CI, 1.3-1.9; *P* = .01) or less than 52 days of past-year marijuana use (RR, 1.7; 95% CI, 1.2-2.4; *P* = .006) (Table 5). After controlling for the same covariates as in the final model for young adult offspring’s marijuana use plus parental alcohol use status, risk of past-month binge alcohol use was higher among young adult offspring whose fathers had lifetime marijuana use (ARR, 1.3; 95% CI, 1.1-1.6; *P* = .02).

## Discussion

This cross-sectional study found substantial prevalence of marijuana use among US parents living with offspring aged 12 to 30 years, in that 7.6% to 9.6% of parents reported past-year marijuana use, and 3.5% to 5.4% of parents used marijuana on 52 days or more in the past year. Although parental marijuana use is reportedly lower than in nonparenting populations,^34^ this protective factor may be reduced with increasing marijuana use among parents.^35,36^

Consistent with previous research showing increased marijuana use in adolescent offspring of parents who use marijuana or have a marijuana use disorder,^22,37,38,39^ our study provides further evidence on associations of parental marijuana use with offspring substance use. First, parental past-year marijuana use was consistently associated with increased unadjusted risk of past-year marijuana, tobacco, and alcohol use and opioid misuse among both adolescent and young adult offspring. Second, even if a parent had lifetime (without past-year) marijuana use or relatively less frequent use, unadjusted prevalence of past-year substance use among offspring was generally higher than those whose parents never used marijuana. These results suggest that parental marijuana use is a risk factor for offspring substance use or misuse across a broad range of substances, including marijuana, tobacco, alcohol, and opioids, even when parental marijuana use is less frequent or in the past. Third, to further understand the specificity of the association of parental marijuana use with offspring substance use, we used multivariable models that adjusted for potential confounders related to offspring, familial, and environmental factors and found that parental marijuana use is a specific risk factor for marijuana and tobacco use by adolescent and young adult offspring and for alcohol use by adolescent offspring. Fourth, adolescent offspring’s substance use appeared to be particularly associated with mother’s marijuana use status. Even after adjusting for potential confounding factors, including the mother’s alcohol use status, the mother’s marijuana use status was associated with the adolescent offspring’s past-year alcohol use. Our results indicate that the mother’s marijuana use status was more than a substance use risk factor and suggest the differential and pivotal roles that a mother plays in the development of her adolescent offspring.

Environmentally mediated normalization of substance use and increased access to substances at home are possible explanations.^40,41,42,43,44^ However, we were unable to further explore mechanisms underlying parent to child transmission because the NSDUH did not capture drug potencies, preparation type and route, whether use was predominantly at home, in the presence of children, or whether offspring had access to or handled marijuana at home.^40^ Modulators of offspring use reportedly include the sex of the parent and the child, children’s ages, family dynamics, sibling and peer influences, and parental involvement or neglect.^14,15,16,40,41,42,43,44,45,46,47^ Regardless of mechanisms, clinicians and parents should be aware of the significant influence of parenting, parental marijuana use, and the poor prognosis associated with early marijuana initiation.^48^ Substance use prevention among adolescents is a public health imperative because early initiation of marijuana use is associated with more severe symptoms and consequences,^4,5,6,7,8,9,11,12,13^ including marijuana and other substance use disorders and comorbidities, such as affective, anxiety, and personality disorders.^49^

Given the poor prognosis of substance use disorders for long-term outcomes, direct and indirect screening in medical settings of family members for marijuana use is an important and achievable goal endorsed by the US Preventive Services Task Force.^50,51^ A positive screening result should trigger counseling of parents on risks posed by using and storing marijuana, tobacco, or opioids at home, educating parents on risk and protective factors, and offering reassurances that substance use is modifiable.^52,53^ Reducing marijuana, tobacco, and other substance use by offspring living with a parent using substances is approachable by targeted strategies, such as strengthening families,^54^ or with universal prevention approaches that combine mass media campaigns adapted from tobacco cessation campaigns, including school- and community-based programs and changing statewide or community-wide policies and norms.^55^

### Limitations

This study has several limitations. The NSDUH excluded people experiencing homelessness who were not living in shelters, which may underestimate associations between parental marijuana and tobacco use and substance use patterns of their offspring because families experiencing homelessness often have a higher prevalence of substance use compared with the general population.^56^ The NSDUH did not sample offspring and both parents in any households and cannot provide insights into full patterns of family substance use. Nor did the NSDUH collect lifetime or past-year binge and heavy alcohol use; thus, we cannot control for these factors in our analyses. Information is unavailable about specific patterns of marijuana use by parents and offspring (eg, use together, use in the home, types of marijuana products used). Moreover, because of the cross-sectional nature of NSDUH data and related limitations of NSDUH questionnaires, this study could not establish temporal or causal relationships. The NSDUH are self-reported surveys and subject to recall and social desirability biases.^57^ Furthermore, the 2015 through 2018 NSDUH did not collect the biological, step, adoptive, or foster relationship data between sampled parent and offspring. However, based on the 2014 NSDUH data, among adolescents aged 12 to 17 years living with a mother who was born from 1955 to 1984, 94.2% reported that their “mothers” were their biological mothers. Additionally, we may not have considered all confounding factors in our multivariable analyses. Future research is needed to confirm the significant associations identified in our study and to explicate these findings with details about patterns of marijuana use by parents and offspring.

## Conclusions

This cross-sectional study found that parental recent and past marijuana use was associated with an increased risk of marijuana and other substance use by adolescent and young adult offspring living in the same household. As any substance use among young people increases the probability of using other or multiple psychoactive substances and of experiencing substance-related consequences,^58,59^ preventing a cycle of multigenerational substance use should be a national priority.
